# Structure of the human chromosome interaction network

**DOI:** 10.1371/journal.pone.0188201

**Published:** 2017-11-15

**Authors:** Sergio Sarnataro, Andrea M. Chiariello, Andrea Esposito, Antonella Prisco, Mario Nicodemi

**Affiliations:** 1 Institut de Génétique et de Biologie Moléculaire et Cellulaire, 67404 Illkirch, France; 2 Dipartimento di Fisica, Universitá di Napoli Federico II, and INFN Napoli, CNR-SPIN, Complesso Universitario di Monte Sant’Angelo, 80126 Naples, Italy; 3 CNR-IGB, via Pietro Castellino 111, Naples, Italy; 4 Max Delbrück Center for Molecular Medicine in the Helmholtz Association (MDC), Berlin, Germany; Parc de Recerca Biomedica de Barcelona, SPAIN

## Abstract

New Hi-C technologies have revealed that chromosomes have a complex network of spatial contacts in the cell nucleus of higher organisms, whose organisation is only partially understood. Here, we investigate the structure of such a network in human GM12878 cells, to derive a large scale picture of nuclear architecture. We find that the intensity of intra-chromosomal interactions is power-law distributed. Inter-chromosomal interactions are two orders of magnitude weaker and exponentially distributed, yet they are not randomly arranged along the genomic sequence. Intra-chromosomal contacts broadly occur between epigenomically homologous regions, whereas inter-chromosomal contacts are especially associated with regions rich in highly expressed genes. Overall, genomic contacts in the nucleus appear to be structured as a network of networks where a set of strongly individual chromosomal units, as envisaged in the ‘chromosomal territory’ scenario derived from microscopy, interact with each other via on average weaker, yet far from random and functionally important interactions.

## Introduction

New technologies, such as Hi-C, can measure the frequency of physical contacts between DNA segment pairs genome-wide and are revealing the complex spatial organization of the mammalian genome [1–4]. A complex network of interactions exists, having functional roles as, for instance, regulatory regions, such as enhancers, often control gene expression at long genomic distances through the formation of loops with their target genes based on physical interactions. Network analyses of Hi-C contacts between DNA segments have provided several insights on chromatin organization, including the identification of their enrichment for genes and chromatin marks, the presence of gene hubs and the possibility to reconstruct 3D conformations of genomic loci (see, e.g., [5–7] and ref.s therein). It has been discovered, in particular, that each chromosome is composed by a sequence of domains (also named Topological Associated Domains, TADs [8, 9]) that are enriched for internal interactions with respect to background [8, 9] and have a hierarchy of long range contacts [10]. Identified by different computational methods (see, e.g., ref.s [8–11]), they are partially conserved across cell types.

To derive a large-scale picture of the structure of nuclear organization of human chromosomes, here we investigate specifically the interaction network of the *contact domains* defined by Rao and others [11] from in-situ Hi-C data in human GM12878 cells (see Methods—Contact Domains): contact domains are identified by the *Arrowhead algorithm* that scores the DNA regions of sites having similarly enhanced levels of Hi-C mutual interactions and, correspondingly, a drop in contacts at their borders. We focus on contact domains as they represent single architectural units and are marked by coherent epigenetic signatures. Additionally, they have a median length of 185kb (which sets the genomic resolution of our study) that is one order of magnitude larger than the size of genes, providing stability to our analysis against the noise inherent to long-range Hi-C data, without compromising too strongly on resolution.

**Fig 1:**
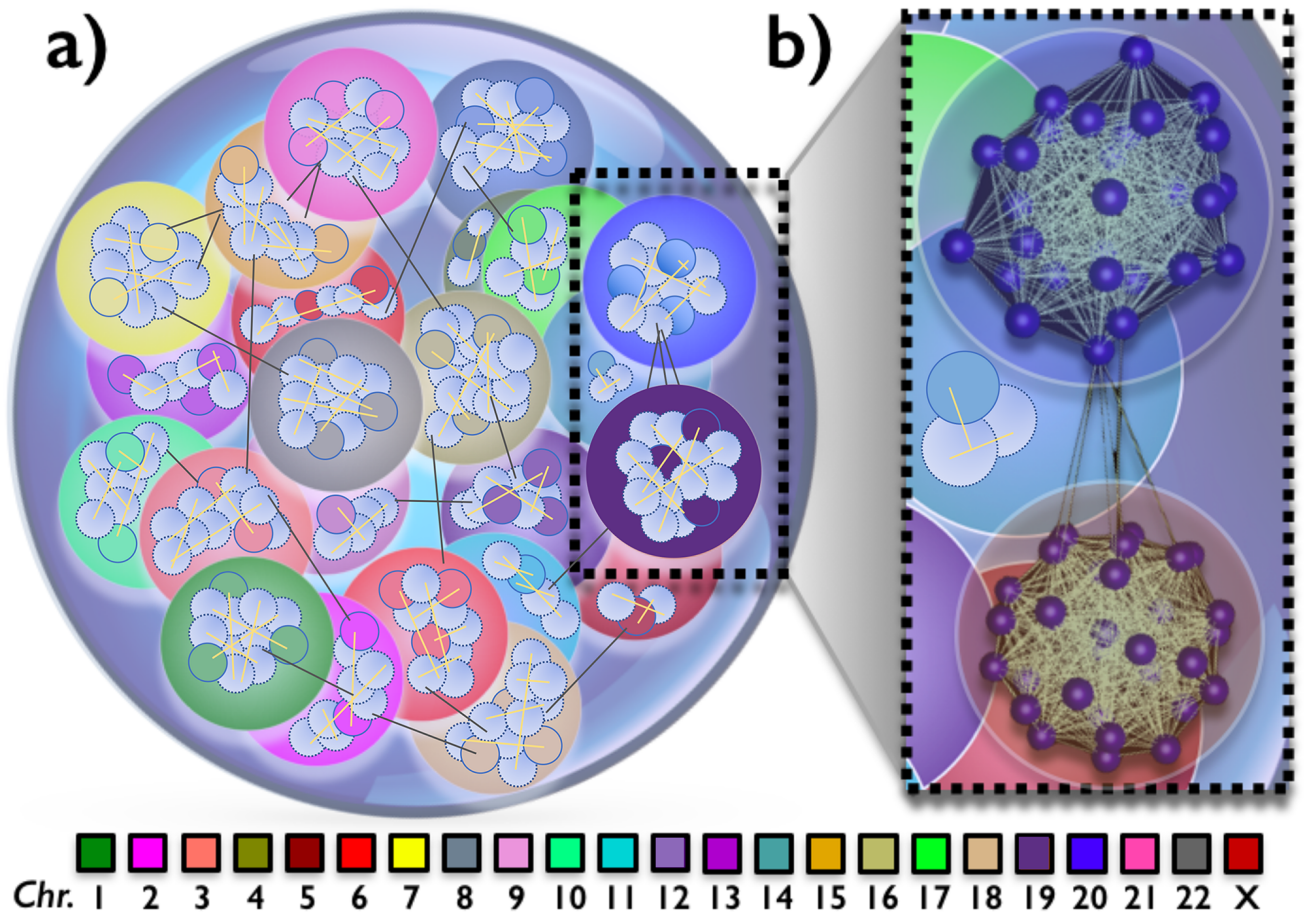
Our analysis of Hi-C genomic interactions shows that the nucleus of human GM12878 cells is structured as a net of networks. Each individual chromosome form a strong intra-chromosomal network of contacts, consistent with the ‘chromosomal territories’ seen by microscopy. Yet, chromosomes intermingle via weaker, yet non-random and functionally important contacts, whereby distinct chromosomal networks form a global nuclear network. Panel a: A pictorial representation of the 3D organization of chromosomes (colored spheres) within the nucleus, as emerging from our network analysis. Panel b: A real network reconstruction (using the visualization tool described in [14]) of chromosomal contacts of chromosomes 19 and 20. For clarity of presentation, each sphere inside a chromosome represents ∼ 1*Mb* (5 contact domains) and a high threshold is set for link visualization.

We find that their intra-chromosomal contact networks are marked by a ‘universal’ power law distribution of interaction frequencies. Inter-chromosomal contacts are found to be approximateley three orders of magnitude weaker [1, 11], yet, we show that they are also far from randomly positioned along the genome. We find that their network is structurally different from the net of intra-chromosomal interactions, as the intensity of contacts is exponentially distributed. Interestingly, intra-chromosomal contacts are typically enriched between epigenomically similar domains, whereas inter-chromosomal contacts are found especially between domains rich in highly expressed genes. The picture of the nucleus emerging from our analysis (schematically shown in Fig 1) returns a scenario where chromosomes contacts form a net of networks. Single chromosomes are mainly folded in territorial units, as seen by microscopy [12], having a complex internal organization. Different territories, though, interact with each other in a non-random, functional way, as envisaged in the ‘chromosome intermingling’ scenario, where chromosome pairs intermingle as revealed by in situ-hybridization technique (cryoFISH) [13].

## Results

### The system

In our analysis, the nodes of the chromosome interaction network are the contact domains, while the edges between nodes are defined by the intensity of their interactions. Human GM12878 cell chromosomes are divided in 9274 contact domains, having an exponentially distributed genomic length with a median equal to 185kb, covering roughly 70% of the entire genome [11]. They have been grouped in six categories, named ‘subcompartments’, A1, A2, B1, B2, B3, B4, having a median length of 300kb, which correlate with specific epigenetic patterns [11]. Subcompartments A1 and A2 belong to ‘compartment A’ [1] and are rich in highly expressed genes and activating chromatin marks. A1 and A2 exhibit early replication times, with A2 having lower GC content, longer genes and stronger association with the H3K9me3 histone mark than A1. B1, B2, and B3 are associated with ‘compartment B’: B1 correlates with facultative heterochromatin, B2 with pericentromeric heterochromatin and B3 with nuclear lamina. B4 is present only in chromosome 19 and correlates with both activating chromatin and heterochromatin. Genomic regions within a single contact domain also have correlated histone marks, while 66,5% of contact domains overlap with a single subcompartment.

The interaction between two domains is defined as the sum of their shared entries in the normalized in-situ Hi-C contact frequency matrix divided by the product of their genomic lengths (see Methods—Contact Domains). Some contact domains are contained within another contact domain. Albeit they represent only a fraction of the total (∼35%), in our analysis we disregard them to avoid double counting of interactions. However, we checked that our results remain unchanged if the entire set in considered. The median length of the remaining domains is 250kb. Based on in-situ Hi-C data, the ratio of the average interaction strengths of domain pairs respectively on the same and on different chromosomes is *I*_*in*−*cis*_/*I*_*in*−*trans*_ = 850, showing that the mean interaction within a chromosome is almost three orders of magnitude higher than across chromosomes [11]. Hence, we initially focus on intra-chromosomal interaction networks.

### Intra-chromosomal interaction networks

To characterize the structure of inter-chromosomal contacts, we first measured the node average normalised degree of connectivity, *n*. This quantity is defined as the number of domains linked to a given domain on the same chromosome, normalized to the total number of domains on that chromosome. To establish whether two nodes are linked, we check if their interaction level is higher than a chosen minimum threshold, in order to avoid spurious effects related to noise in the data. A typical way to identify significant interactions [10] is to retain only those that fall above a threshold, so to filter the noise due to random interactions; here, we consider as threshold the value corresponding to the lowest 25-percentile of the distribution of inter-chromosomal domain interactions. The rationale behind such a choice is that measured contacts between pairs of domains on different chromosomes are more likely to be random, also considering the vastly higher number of possible pairs for the experimentally set sequencing depth. For such a set threshold we find *n* = 0.998. To check that different threshold values do not alter our scenario, we measured that when the 75-percentile is considered we get *n* = 0.979. The order of magnitude of *n* is unchanged even when the threshold is set based on percentiles of the distribution of intra-chromosomal interactions. Hence, at the considered genomic resolution, we find that the domains of a single chromosomes form a fully connected network, where approximately all nodes interact with all. That points out the highly dynamical nature of chromatin contacts.

**Fig 2:**
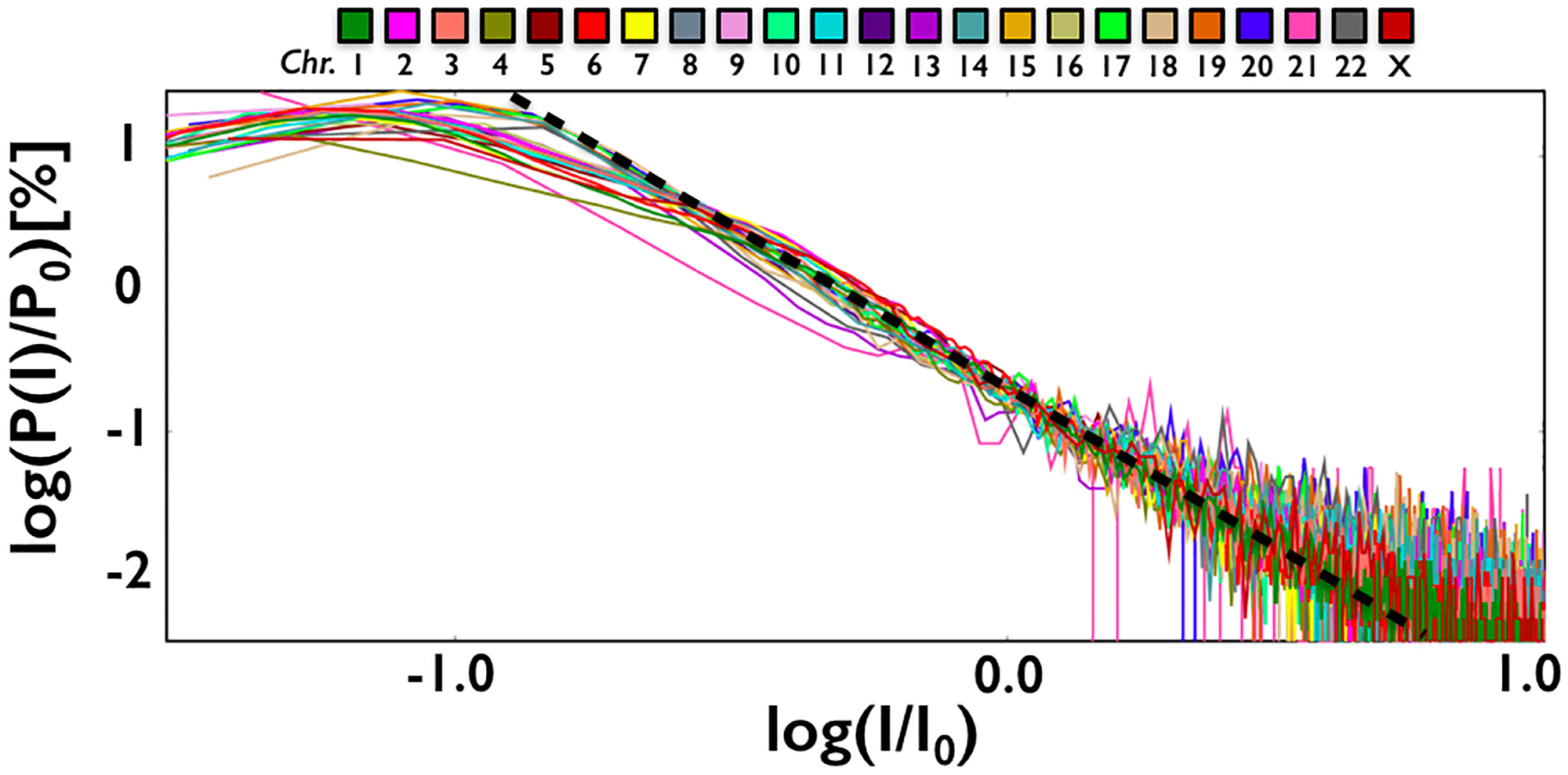
The distribution of intra-chromosomal interactions between contact domains (here in a *log*_10_ − *log*_10_ plot) decays as a power law, *P*(*I*) ≃ *I*^−*γ*^, with an exponent close to *γ* ≃ 2 (dashed line), which appear to be ‘universal’ across different chromosomes. So, the structures of their contact networks exhibit similar quantitative features. *I*_0_ and *P*_0_ are factors to rescale on top of each other the data from different chromosomes.

We focused next on the distribution of the intensity of intra-chromosomal interactions, *P*(*I*). We found that it has a power law decay over roughly two orders of magnitude, *P*(*I*) ≃ *I*^−*γ*^ (Fig 2). The exponent is close to *γ* ≃ 2 and very similar in different chromosomes. This is an interesting observation because an exponent *γ* = 2 marks scale free networks [15], which have a special relevance as they are characterized by the presence of *hubs*, i.e., nodes with very high levels of contacts. To test the statistical significance of our analysis, we used the python library *powerlaw*, a package for the analysis of heavy-tailed distributions [16]. For every chromosome, we found that the tail of the distribution fits the best with a power law, with a negligible *p* − *value*. This points out that contact frequencies can extend to very large values for some genomic regions. Interestingly, the distribution, *P*(*I*), and its exponent *γ* ≃ 2 are ‘universal’ across chromosomes, hinting that the structure of their domain networks is similar, as shown in Fig 2.

**Fig 3:**
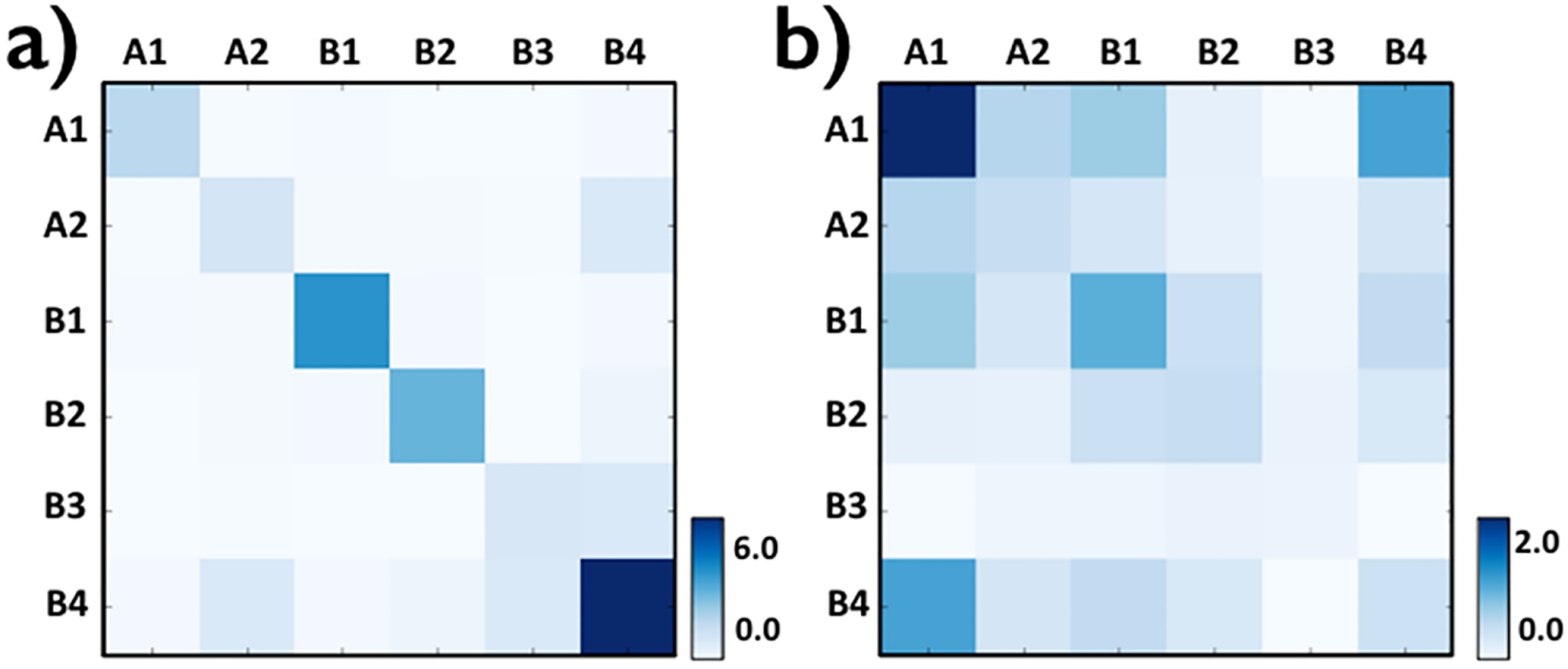
Heatmaps representing, across the different subcompartments A1-B4, the domain average excess interactions over background within single chromosomes (*in-cis*, Panel a) and between chromosomes (*in-trans*, Panel b). Intra-chromosomal contacts (a) especially occur between homologous subcompartments (diagonal), while inter-chromosomal contacts (b) are particularly frequent with subcompartment A1, rich in highly expressed genes, hinting to a functional role.

To help dissecting the molecular nature of the determinants of contacts, we then investigated the enrichment of intra-chromosomal domain interactions across the different subcompartments, which are known to be associated each with a different set of epigenetic features [11]. In our analysis we consider only domains overlapping with a single subcompartment (77% of total), but our conclusions stay unchanged if all domains are considered and each one is assigned to a single subcompartment, e.g., by a majority rule. We repeated our analysis also for the top interacting domains, i.e., those in the top 5% of *P*(*I*) distribution (see Methods—Chromosomal domain interactions across subcompartments). While single domains are prevalently overlapping with A1, followed by A2 and B1 [1], we found that the top interacting ones are more evenly spread across the different subcompartments (see Methods—Contact domain distribution across subcompartments). The heatmap in Fig 3, Panel a, reports the average interaction between domains in different subcompartments, normalized by the overall average value. Interestingly, enrichments of contacts are mainly seen along the diagonal, showing that intra-chromosomal contacts tend to be homotypical, i.e., usually occur between epigenomically homologous regions. In particular, the strongest homotypic interactions are found within the heterochromatin B1 and B2 subcompartments, and to a minor extent within A1 domains that are rich in active genes. The lamina linked B3 subcompartment has, instead, comparatively lower levels of homotypic interactions. Similar results are found when only the top interacting domains are considered (see Methods—Chromosomal domain interactions across subcompartments).

### Inter-chromosomal interaction networks

Next, we turned to the inter-chromosomal interaction network. Interestingly, it results to be also fully connected, albeit less dense than intra-chromosomal nets as its normalised average degree of connectivity is *n* = 0.749 in the case where the threshold is set, as above, to the 25th-percentile of the distribution, moving down to *n* = 0.249 for a 75th-percentile threshold.

**Fig 4:**
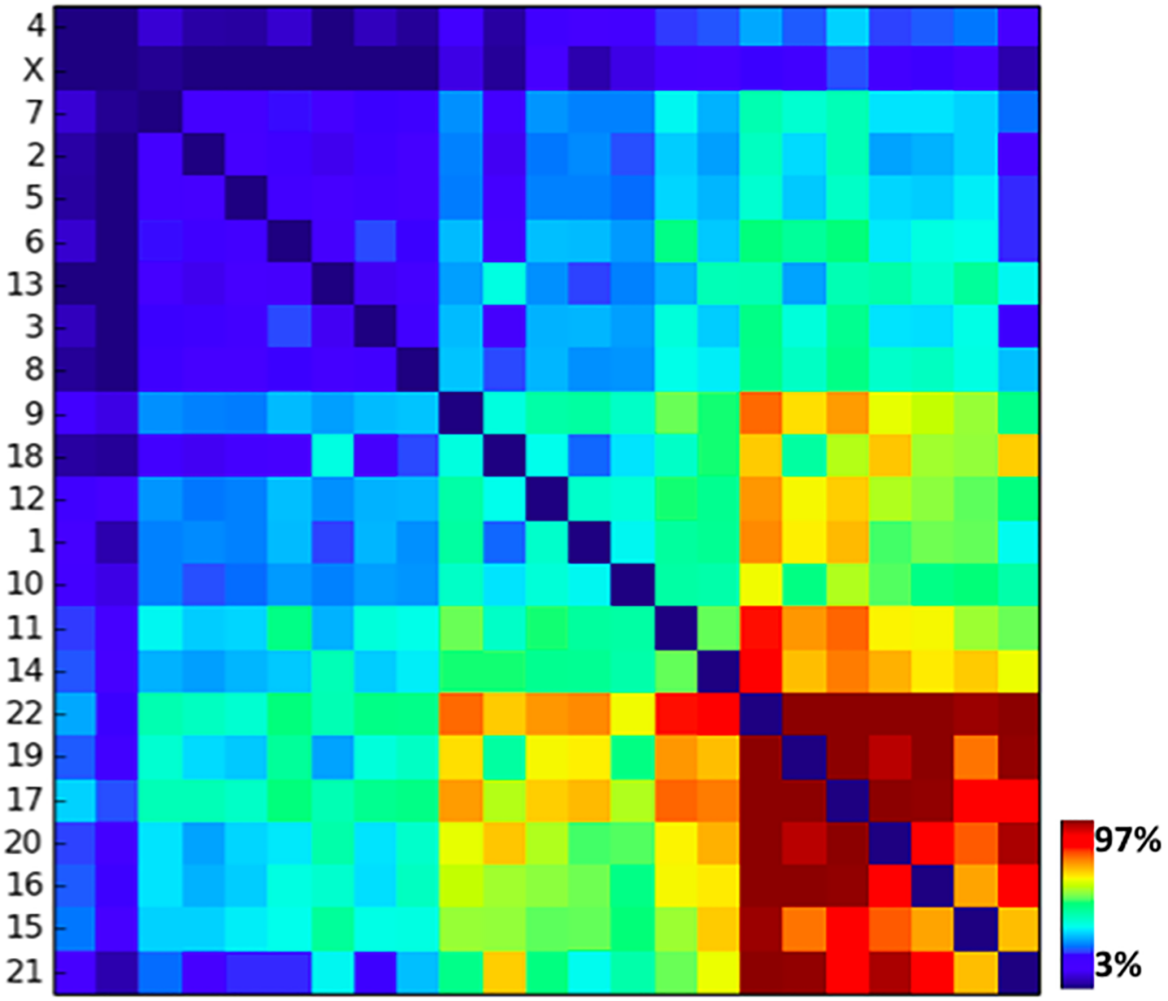
The heatmap of the overall contacts between chromosome pairs. While different chromosomal pairs have different degrees of interactions, the RSS analysis points out that there are no significant isolated subgroups and the system forms a single nuclear network.

To understand whether there are pairs of chromosomes more strongly interacting than others, we investigated their overall pairwise interactions (normalized by their genomic lengths). The heatmap in Fig 4 shows indeed that, as expected, different pairs have different degrees of contact intensities. In particular, gene richer, shorter chromosomes tend to have higher relative reciprocal interactions, consistent with the role of A1 subcompartment in establishing homotypical contacts (see below). To identify whether there are particularly isolated clusters of interacting chromosomes, we followed a standard procedure using the RSS (Residual Sum of Square) objective function [17]. More precisely, we plot the RSS quantity as a function of number of clusters k and seek for the value where the curve has a sharp slope variation (elbow method). From this analysis, we found that there are no evident preferentially isolated clusters at the length scale of contact domains (≃ 200*kb*). Hence, while different chromosomal pairs can have different degrees of interactions, a global, single main inter-chromosomal network exists.

Interestingly, a cross-analysis of chromosomal interactions with the sub-compartment membership (Fig 3, Panel b) reveals that domains belonging to sub-compartment A1 tend to interact mostly. Since sub-compartment A1 is rich in highly expressed genes, that suggests a functional role of highly interacting nodes in the inter-chromosomal network, in agreement with previous findings ([18]). Furthermore, as discussed below, a non trivial pattern of interaction exists.

**Fig 5:**
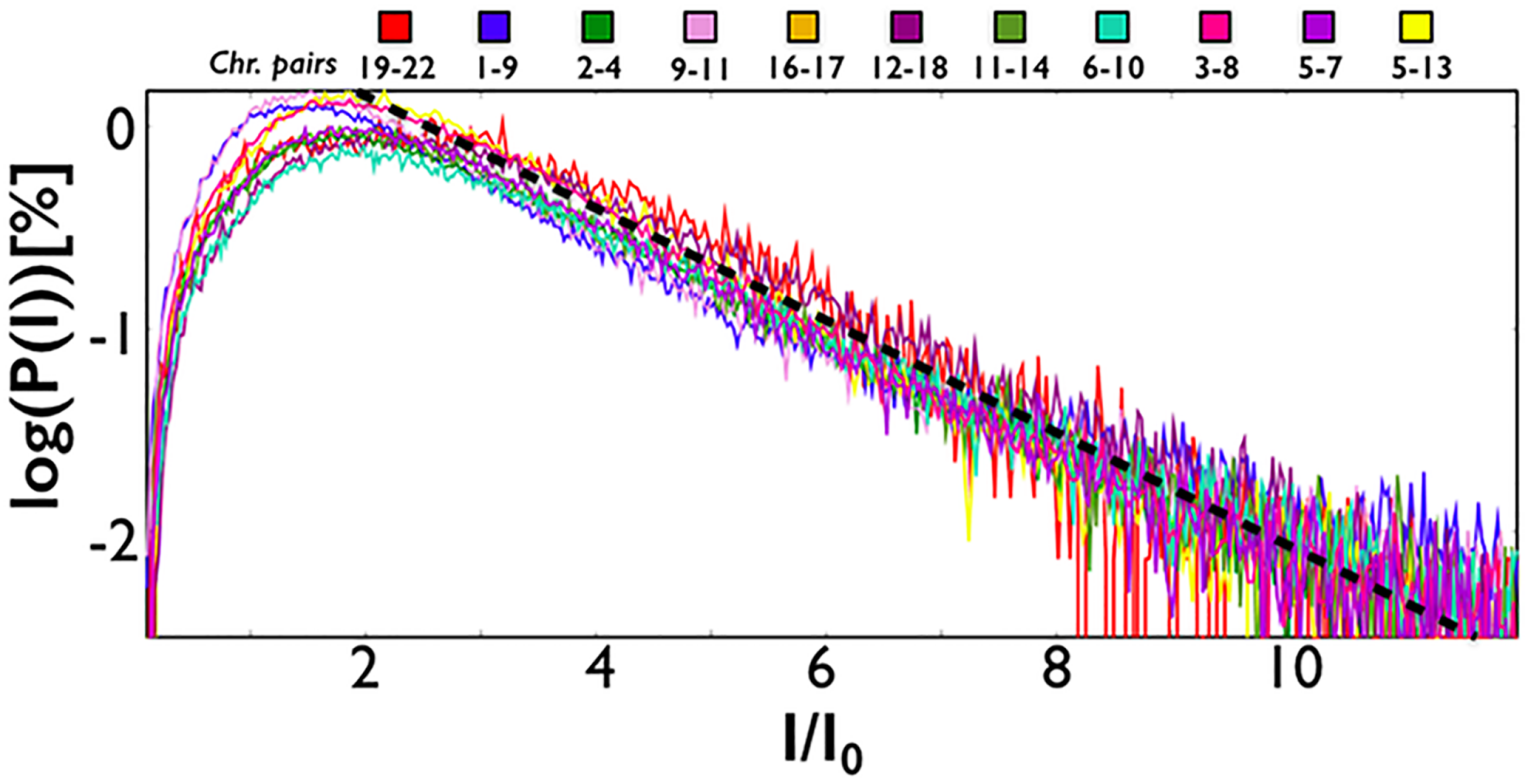
The distribution of interactions between contact domains on different chromosomes (here in a *lin* − *log*_10_ plot) has an exponential decay (dashed line) across chromosome pairs. That shows that the inter-chromosomal network of contacts is structurally different from the power-law distributed intra-chromosomal networks (Fig 2).

The distribution of the intensity of inter-chromosomal interactions, *P*_*inter*_(*I*), is similar across different chromosome pairs and has roughly an *exponential* decay, *P*_*inter*_(*I*) ≃ exp (−*I*/*I*_0_) (Fig 5), as would be expected for a random network [15]. To statistically prove the robusteness of this result, we fitted the tail of the distributions with an exponential function and with a power law function. We performed the analysis using the *optimize* python package. For every possible pair of chromosomes we obtained a coefficient of determination *R*^2^ higher in the exponential case than in the power law case, and extremely close to 1 (average *R*^2^ = 0.989). Moreover, in the 26% of the cases, the power law fit was not convergent. As additional check, to test if intra- and inter-chromosomal distributions were statistically different, we performed the same analysis on the intra-chromosomal datasets, finding a coefficient of determination *R*^2^ sistematically higher for the power law fit than for the exponential one. Thus, we can conclude that the two distributions are not compatible, suggesting that the inter- and intra-chromosomal contact networks are structurally different.

**Fig 6:**
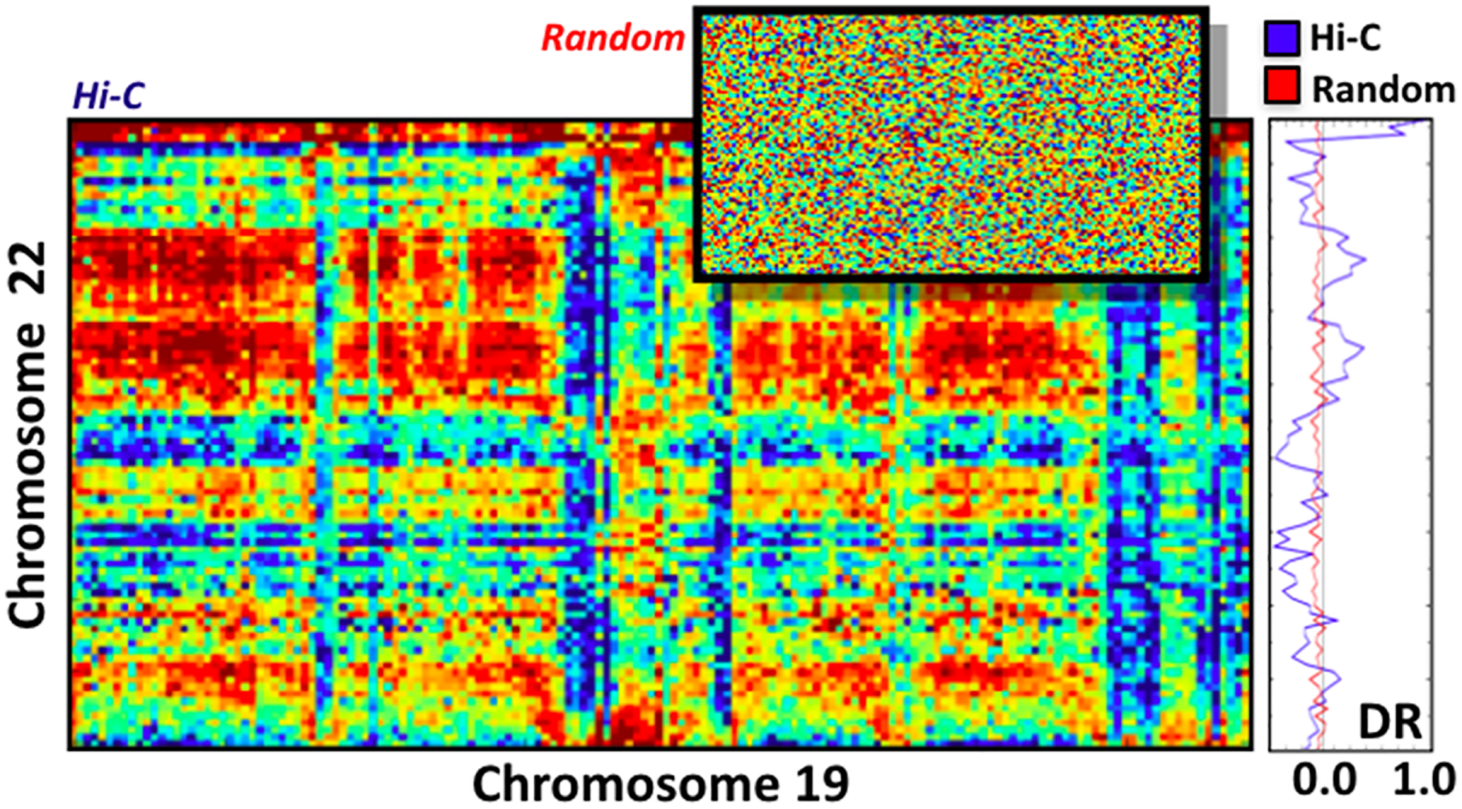
Heatmap of the normalized in-situ Hi-C interactions between the contact domains of chromosomes 19 and 22. Bands of regions with above background signal are visible, in contrast to the corresponding random matrix (top-right, see details in the text). The differential interaction score, *DR*, of the domains of chromosome 22 with 19 (rightmost panel) quantifies the dissimilarity with the random control and highlights the clustering of genomic locations enriched in contacts with domains on the other chromosome.

However, we find that inter-chromosomal interactions are not randomly distributed over the genomic sequence. This is visible in the example of Fig 6 showing a heatmap with the frequency of contact between the domains of chromosome 22 v.s. 19: genomic bands with markedly strong interaction are seen, pointing out that regions enriched for contacts tend to cluster along the chromosomal sequence. For comparison, we also show a random contact heatmap generated by mixing the positions of the values in the experimental matrix so that the distribution of the intensities is unaffected.

To quantify such observations, we computed the differential interaction score, *DR*, across the domains of each single chromosome. For a given domain *k*, *DR*_*k*_ is defined as follows: *DR*_*k*_ = (*S*_*k*_ − < *S* >)/ < *S* >, where *S*_*k*_ is the sum of its contacts with all the other domains of the other considered chromosome (i.e. the sum of rows in the matrix of Fig 6), and < *S* > is the average over the matrix (see Methods—The differential interaction score, DR). The *DR* signal of chromosome 22 v.s. 19 is shown in Fig 6-right panel. As expected, *DR* varies around zero, yet its standard deviation *σ* = 0.3 is one order of magnitude larger than in the random bootstrapped case (*σ*_*rand*_ = 0.05, red line), reflecting the much stronger variations present in the real contacts. We also find that the average size of genomic regions where *DR* is coherent in sign spans approximately 5.4 consecutive domains, against 2.5 in the random case, highlighting the clustering of genomic positions enriched in contacts with domains on a different chromosome. Similar results are found across other chromosome pairs.

Finally, as mentioned before, Fig 3, Panel b reports the average inter-chromosomal interaction strength of domain pairs across the different subcompartments, showing that A1 domains are enriched for interactions, especially with homologous domains. As above, similar observations apply when only the top interacting domains are considered (see Methods—Chromosomal domain interactions across subcompartments). The finding that domains in the A1 subcompartment have preferentially homotypic interactions also supports the view that inter-chromosomal contacts are far from random, as random contacts would be equally enriched across subcompartments.

## Discussion

Taken together, our results show that, at the 200kb resolution, chromosomes are characterised by an extended network of contacts. Intra-chromosomal interactions have a similar structure across chromosomes, marked by broad, power-law distributed interaction frequencies with an exponent *γ* ≃ 2 typical of scale-free nets. In facts, scale-free networks are well known in biological systems and are thought to reflect fundamental organizational principles [15].

Chromosomes are also widely interacting with each other, without isolated subgroups, yet their global interaction network is two orders of magnitude weaker in intensity than intra-chromosomal nets, and exponentially distributed. Hence, the net of inter-chromosomal contacts is structurally different from intra-chromosomal ones. A limitation of our study is that the data do not allow to distinguish the different alleles. Our analysis suggests, though, a non-random genomic positioning of inter-chromosomal interactions.

Our analysis of correlations between epigenetic signals and domain contacts, albeit limited to a few general marks, shows that intra-chromosomal interactions tend to occur between homotypic epigenomic regions and inter-chromosomal interactions are especially found between regions rich in highly expressed genes.

The relative position of chromosomes in the nucleus, based on their domain in-situ Hi-C interactions, is shown in Fig 1, Panel a, as produced by a standard tool for the 3D visualization of network graphs [14]. The zoom in Panel b shows, in particular, the reciprocal interactions of chromosome 19 and 20, along with their intra-chromosomal networks (for clarity of presentation, a high threshold is set for link visualization). Multilayer networks, or networks of networks, are thought to be important to process and spread efficiently information across different nets to produce emergent, collective behaviours in complex natural and biological systems [19, 20]. Our analysis suggests a networked organization of chromosomal interactions where the distinct nets of intra-chromosomal contacts are assembled in a weaker, yet non-random global nuclear network of nets. This important feature is known as modularity. Many network systems in nature have strong modular aspects [21]. An important reason explaining such a peculiar behaviour lies in the high stability that those networks exhibit as response to external perturbations, as recently highlighted by important studies [22]. Hence, our finding could hint towards the modular and structurally stable structure of the 3D architecture of the human genome within the cell nucleus. Our early investigations of the network features of chromosomal interactions may raise the attention of the scientific community on such an interesting topic and pave the way to more extensive bioinformatics and computational analyses to better understand the underlying features and mechanisms leading to the observed pattern in the considered framework, as known for other networked systems ([23, 24])

The emerging large-scale picture of chromosome contact networks in the cell nucleus is complemented by current higher-resolution studies employing models from polymer physics, which try to explain the molecular mechanisms shaping the 3D conformation of specific genomic loci [25–31]. Brief reviews of such models can be found in ref.s [32–34]. Our results are derived from human GM12878 cell data, however, Hi-C interaction patterns are broadly conserved across different cell types in higher mammals [2–4, 8–10], hinting towards a broader validity of our conclusions. In particular, the picture emerging from our quantitative investigation of Hi-C data reconciles the so-called “chromosomal territory scenario” [12] with the “chromosomal intermingling sceanio” [13], as in the nuclear network of contacts chromosomes tend indeed to form strong territorial units, but have also relevant inter-chromosomal interactions.

## Methods

### Hi-C dataset

We employed the primary in-situ Hi-C dataset produced by Rao and others [11], relative to human cells, cell line GM12878, MAPQGE30 at 10Kb of resolution. The row data were normalized by the Knight-Ruiz method [35].

### Contact domains

We used the list of contact domains provided by Rao and others [11], obtained by their *Arrowhead Algorithm*. As discussed in the previous sections of the paper, we excluded from our analysis *internal* contact domains, i.e. those completely contained in other contact domains, to avoid double counting of contacts.

**Fig 7:**
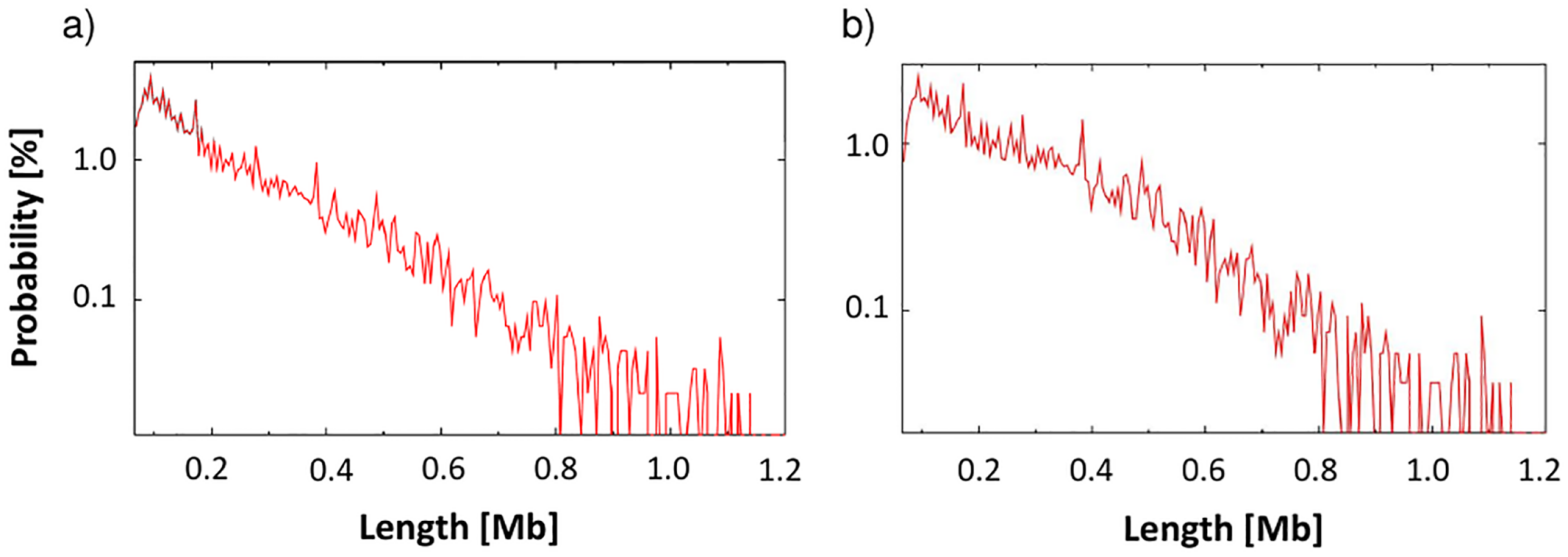
Panel a: Distribution of the lengths of all the contact domains, i.e. the probability to find a contact domains with a given genomic length, represented in a log-lin scale. Panel b: The same analysis for the case where ‘internal domains’ are removed from the list.

In Fig 7 we report the distribution of the lengths of the contact domains, considering all of them (left panel) and removing the internal ones (right panel). We have 9274 contact domains in total, having an average length of 258Kb and a median length of 185Kb. After removing the internal domains, we are left with 5370 contact domains, having an average length of 320Kb and a median length of 250Kb. Different mechanisms of polymer physics [36–40] could contribute to the assembly of contact domains [11, 25–29, 41–43], while additional effects, such as confinement, crowding, entanglement could have a role as found in other complex fluids (see, e.g., [44–53] and ref.s therein).

To analyze the structure of the domain network, we considered the average frequency of contacts between all pairs of domains. The average frequency of contacts, *I*_*a*, *b*_, between domain *a* and *b* is defined as the sum of their shared entries in the normalized in-situ Hi-C matrix divided by the product of their genomic lengths.

### Chromosomal domain interactions across subcompartments

To investigate the enrichment of intra- and inter-chromosomal domain interactions across the different subcompartments, we computed the average interaction between domains in different subcompartments, *H*_*i*,*j*_, normalized by the overall average value. More precisely, the values *H*_*i*,*j*_ in each bin of the heatmaps relative to Fig 3, are obtained by the following expression: *H*_*i*,*j*_ = (< *H*(*i*, *j*) > − *H*_0_)/*H*_0_, where < *H*(*i*, *j*) > is the average frequency of contact between the contact domains belonging to the subcompartments *i* and *j*. Here *H*_0_ = (∑ < *H*(*i*, *j*) >)/*N*_*c*_ is the average value across subcompartments, and *N*_*c*_ is the number of the couples of subcompartments.

**Fig 8:**
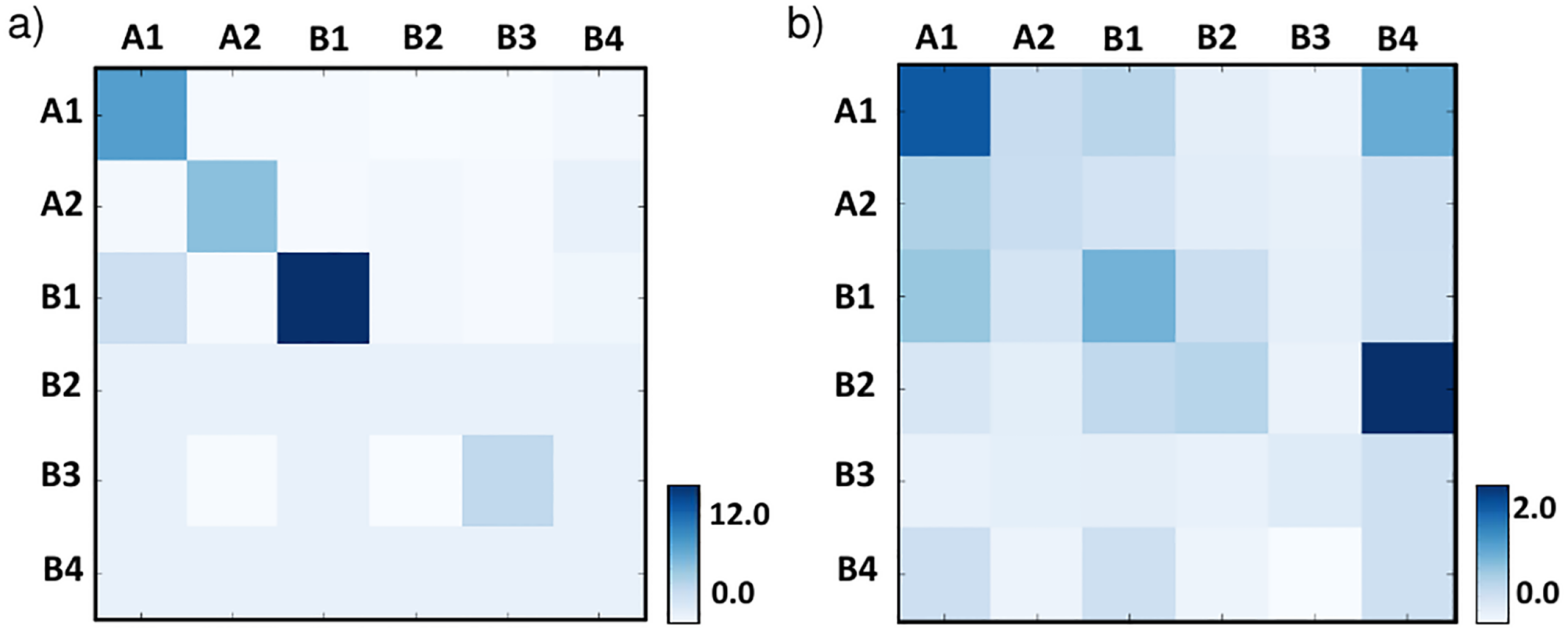
Heatmap representing the average frequency of interactions between ‘top interacting’ contact domains (see text). Panel a: *in-cis* and Panel b: *in-trans*, belonging to the different subcompartments.

We did the same analysis considering only the top interacting contact domains *in-cis* and *in-trans*, i.e. we considered only the 5% of all contacts domains having the highest average frequency of interaction with the other contact domains, respectively on a given chromosome and on an other chromosome (for all the possibile couples of chromosomes). The results are reported in Fig 8

### Contact domain distribution across subcompartments

**Fig 9:**
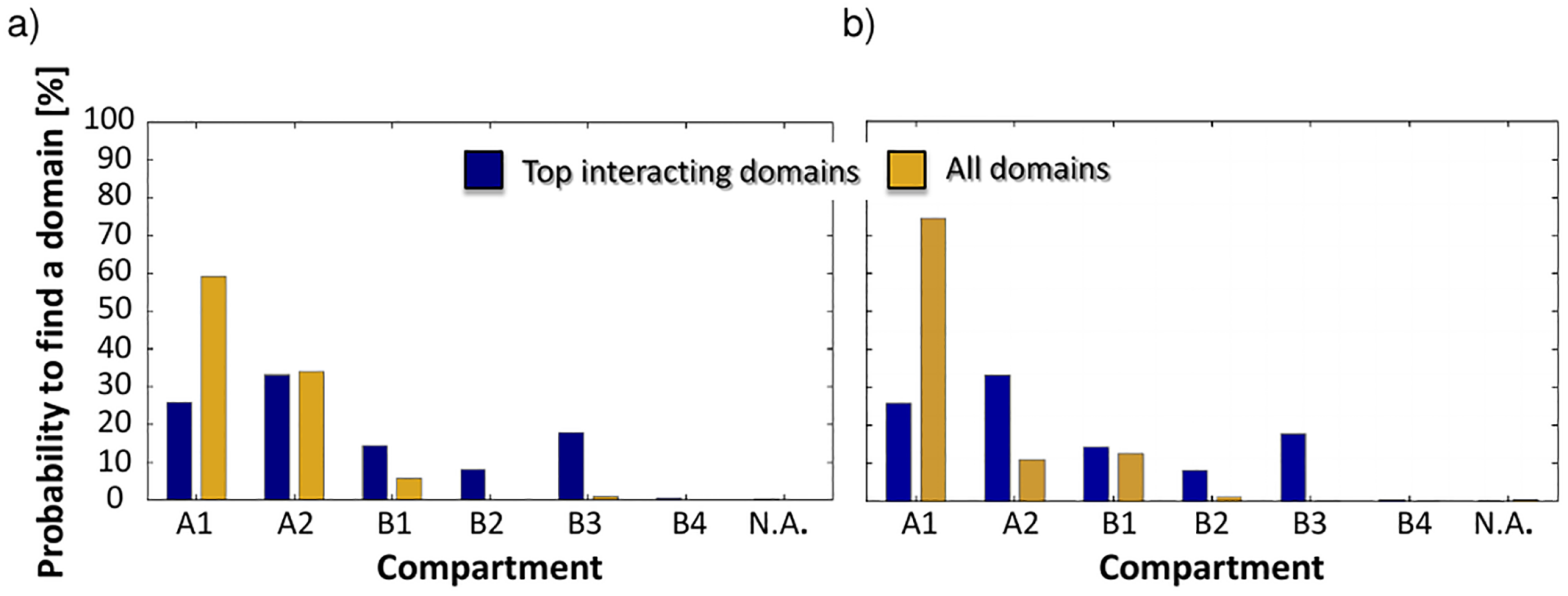
Panel a: Fraction of domains in each subcompartment *in-cis* (N.A. means *Not Assigned*) in two cases: for all domains (yellow) and for the ‘top interacting’ domains in-cis (blue). Panel b: The same analysis *in-trans*.

We also analyzed how domains are distributed across the compartments. In this analysis, we considered the domains overlapping with a single subcompartment (77% of total), but our conclusions stay unchanged if all domains are considered and each one is assigned to a single subcompartment by a majority rule. In Fig 9 we represented two cases: all domains and only the *top interacting domains*, i.e. the 5% of the domain with the higest average frequency of interaction, for *in-cis* and *in-trans* cases (left and right panel respectively). While considering all the contact domains we have a prevalence of domains in compartment A1, with A2 and B1 following, if we consider only the top interacting domains the most abundant group is in compartment A2. Top interacting domains are present in compartment B for about 35%, while there is only a small percentage if we consider all the domains.

### The differential interaction score, DR

The differential interaction score, *DR*, is defined in the in section about inter-chromosomal interactions network. It was computed for all chromosome pairs, i.e., for all the domains of a given chromosome with respect to its interactions with the domains of all other single chromosomes. For example, Fig 6 refers to the *DR*’s of the domains of chromosome 22 in their interaction with those on chromosome 19.

**Fig 10:**
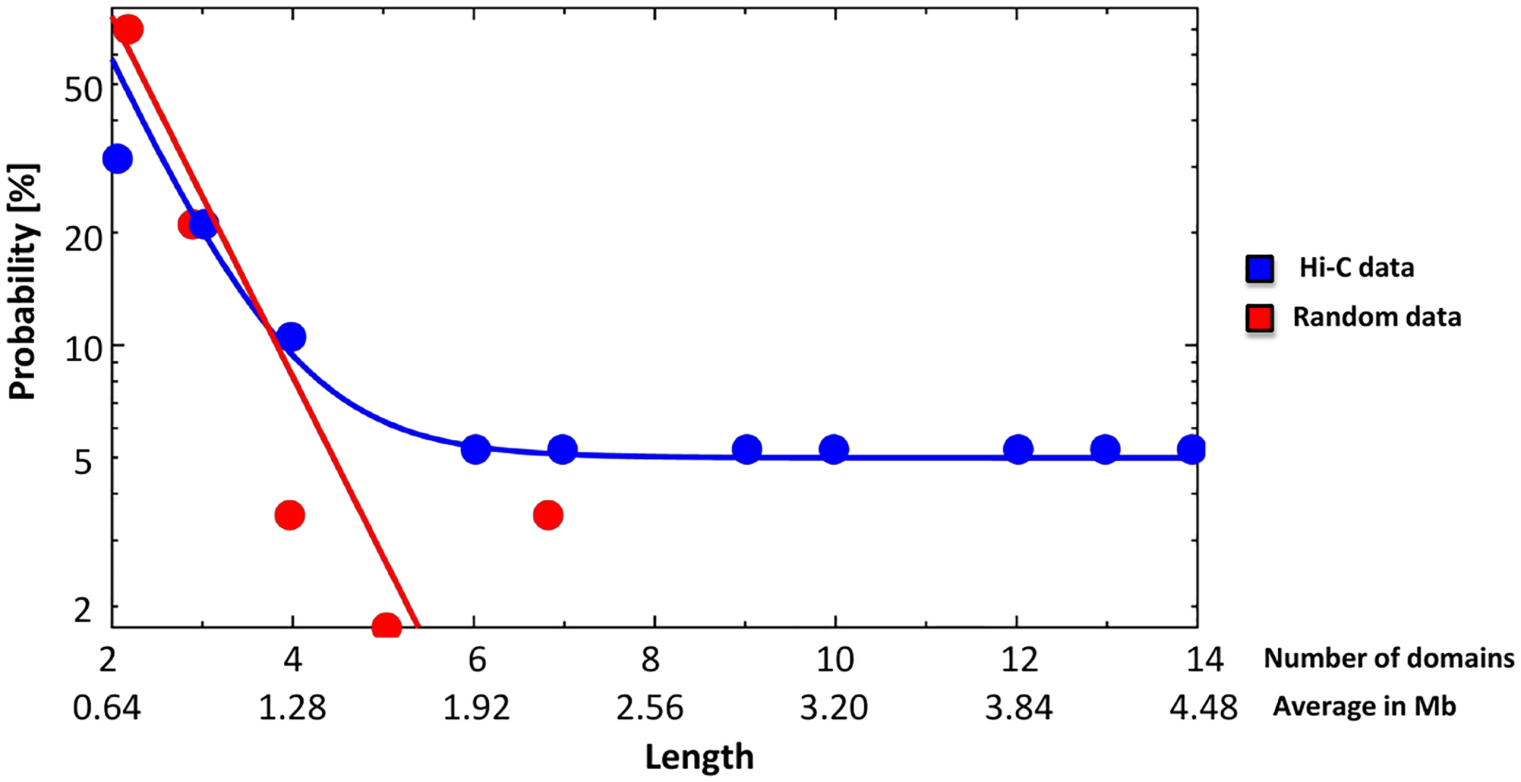
Distribution of the sizes of genomic regions where *DR* is coherent in sign in the random bootstrapped (red) and Hi-C real data (blue), as a function of the number of domains or, correspondingly, in Mb (the average length of each contact domain is 320Kb). The superimposed curves are exponential fits.

Finally, for each chromosome, we also measured the average size of genomic regions where *DR* is coherent in sign, i.e., we calculated the number of contact domains between two consecutive crossings of the zero by *DR*. The distribution of sizes is reported in Fig 10 for the real and the bootstrapped matrices in the case of chromosome 19 v.s 22 discussed in the previous sections of the paper. In both cases an exponential fit is overlaid on the data. Similar findings are obtained for other chromosome pairs.
